# Self-Regulation Behavioural Style and Emotional State in Students and People Living with HIV during the COVID-19 Second Wave in the Russian Federation

**DOI:** 10.1192/j.eurpsy.2025.1886

**Published:** 2025-08-26

**Authors:** V. I. Rozhdestvenskiy, V. V. Titova, I. A. Gorkovaya, Y. S. Aleksandrovich, D. O. Ivanov

**Affiliations:** 1 Department of Psychosomatics and Psychotherapy, Saint Petersburg State Pediatric Medical University, Saint Petersburg, Russian Federation

## Abstract

**Introduction:**

Self-regulation refers to a structured mental process for initiating, organizing, maintaining, and managing internal and external activities directed toward achieving goals. Each individual exhibits a unique self-regulation style that influences their capacity to adapt to changing circumstances. During the COVID-19 pandemic, a significant rise in anxiety, depression, and stress levels was noted in Russia. Emotional disorders may relate to self-regulation styles, as they affect an individual’s adaptation to evolving internal and external stressors.

**Objectives:**

The study investigates relationships between self-regulation styles and depression, anxiety, and stress levels among humanities students and HIV-positive patients, shedding light on how behavioural self-regulation affects emotional responses in different population segments during the pandemic in the Russian Federation.

**Methods:**

Data were collected from January to July 2021 using a Google form. The sample included 35 humanities students from Russian universities and 59 HIV-positive patients. V.I. Morosanova’s “Style of Self-Regulation of Behaviour” questionnaire was used to assess self-regulation styles, while the DASS-21, adapted for Russian contexts, measured levels of depression, anxiety, and stress.

**Results:**

We found that in the group of Russian university students, depression had negative correlations with behavioural programming (r_s_ = -0.421, p < 0.05) and with outcome evaluation (r_s_ = -0.401, p < 0.05). In the HIV patient group, depression had negative correlations with modelling (r_s_ = -0.322, p < 0.05) and flexibility (r_s_ = -0.285, p < 0.05), anxiety also with modelling (r_s_ = -0.270, p < 0.05) and flexibility (r_s_ = -0.261, p < 0.05). In both groups, stress was not related to self-regulation behaviour style.

**Conclusions:**

The study highlights the association between emotional disorders and self-regulation was more pronounced in the HIV-positive group. Depression and anxiety corresponded with reduced reality assessment and flexibility in self-regulation. These patients found it more challenging to evaluate internal and external factors and adjust their self-regulatory processes amid changing conditions, indicating a greater vulnerability to disruptions in self-regulation. Within the students’ group, depression alone affected self-regulation, particularly diminishing abilities in programming and outcome evaluation. As depressive symptoms worsened, students struggled to effectively plan actions and assess their behaviour and achievements.

**Disclosure of Interest:**

None Declared

